# Collaborative Interpretation of Researcher-Generated Photo-Elicitation Findings: Insights From Women With Lived Experience of Homelessness

**DOI:** 10.1177/10497323231224330

**Published:** 2024-01-18

**Authors:** Gustav Bockgård, Elisabet Mattsson, Louise von Essen, Anna Klarare

**Affiliations:** 1 Department of Scandinavian Languages, 8097 Uppsala University, Uppsala, Sweden; 2 Department of Women’s and Children’s Health, Healthcare Sciences and e-Health, 8097 Uppsala University, Uppsala, Sweden; 3 Department of Health Care Sciences, Marie Cederschiöld University, Stockholm, Sweden

**Keywords:** homelessness, photo-elicitation, researcher-generated photos, women, qualitative

## Abstract

Interviews with individuals experiencing homelessness can be challenging for various reasons, including mental and physical health issues, substance use, and negative experiences with authority figures. Researchers have used photos to facilitate communication and empower participants during data collection. We analyzed data from a previous study to explore the use of researcher-generated photos during interviews about health with 13 women experiencing homelessness. Conversation analysis revealed clear patterns regarding the use of the photos during the interviews. The photos were referred to 118 times over the total interview length, 6 hours and 23 minutes, with the interviewer making 62% of the referrals and the women accounting for 38%. Fifty-nine percent of the referrals occurred within the first 5 minutes of the interviews. The women used the photos to trigger associations and emotions, to describe photo content, or in a minor role during the interview. Interpretations from an advisory board of six women with lived experiences of homelessness suggested that the photos did not engage participants as intended, highlighting the importance of considering participants’ perspectives when designing photo-elicitation methods. The feedback also provided valuable insights into interview locations and incentives in research that may have influenced the women’s willingness to use the photos. This study emphasizes the importance of understanding the complexity of choosing researcher-generated photos in interviews with underserved, hard-to-reach populations.

## Introduction

Interviews, guided by distinct theoretical frameworks, are a prominent method for data collection among diverse qualitative researchers (Roulston, 2006). In the mid-1980s, Briggs (1986) estimated that approximately 90% of social science research relied on interviews as a foundational methodology. The etymology of the term *interview*, rooted in the French word *entrevoir* meaning “to see one another,” highlights the fundamental nature of an interview as a mutual exchange of perspectives and information between individuals.

However, interviews with people experiencing homelessness can be complex and challenging for several reasons. Firstly, individuals experiencing homelessness often face a range of physical and mental health issues including substance use (Aldridge et al., 2018; Fazel et al., 2014), making it difficult for them to engage in an interview process. Secondly, they may have negative experiences with authority figures and institutions (Kneck et al., 2021; Omerov et al., 2019), making them wary of participating in research or being interviewed. In addition, individuals experiencing homelessness face stigma and discrimination (Kneck et al., 2022), which may impact their willingness to participate in research or be open and honest during an interview. Consequently, it is reasonable to posit that the conventional research interview setting may inadvertently reinforce hierarchical dynamics and perpetuate a sense of inadequacy for this population, given the inherent power differentials in social standards and resources (Seitz & Strack, 2016). To address these challenges and empower participants in the data collection process, studies have turned to the utilization of visual aids, such as photos or images, as facilitative tools for communication during research interviews (Seitz & Strack, 2016; Wang & Burris, 1997).

The terms photovoice and photo-elicitation are used interchangeably; however, aiming for method stringency, credibility, and trustworthiness, defining terms is called for (Pauwels, 2015). Photovoice is a participatory research method in which individuals are asked to take photos that depict their experiences, perspectives, and issues related to a specific topic (Wang & Burris, 1997). The photos and accompanying narratives are then, through group discussions, used to give voice to the perspectives of the individuals to generate insights and ideas for social change. Photo-elicitation, a core component in photovoice (Wang & Burris, 1997), is a qualitative, research method in which photos are used as a tool to evoke discussion, emotions, information, or memories within a semi-structured interview (Banks & Zeitlyn, 2015; Collier, 1957; Harper, 2002). The goal of photo-elicitation is typically to gain a deeper understanding of an individual’s experiences or perspectives rather than to generate ideas for social change. Two primary variations of photo-elicitation exist, and both involve photos related to the research setting: either participant-generated photos or researcher-generated photos (Richard & Lahman, 2015). In the present study, we use researcher-generated photo-elicitation. We make the distinction that photo-elicitation focuses on the interview process itself, whereas photovoice is an action-oriented research strategy (Bugos et al., 2014).

Harper (1994) and Collier (1995) argue that photo-elicitation can foster a collaborative dialogue between the participant and the researcher to provide a way to account for the context of the research. Furthermore, the literature suggests that the method has the potential to specifically alleviate interview fatigue (Harper, 2002), bridge the gap of different backgrounds (Harper, 2002), create a sense of familiarity in the interview situation (Collier & Collier, 1986), foster exploration and extended responses as well as empowering interactions (Banks, 2007), overcome low literacy/illiteracy (Collier & Collier, 1986), promote participant agency (Banks & Zeitlyn, 2015; Collier & Collier, 1986), and provide interview focus (Banks & Zeitlyn, 2015; Collier, 1995; Harper, 1994). However, photo-elicitation requires a high level of trust between the researcher and the participant, and this trust can be difficult to establish (Harper, 1994). Furthermore, while photo-elicitation can be a powerful tool for eliciting emotions and memories, researchers must be mindful that it may trigger negative emotions or traumatic memories, particularly when dealing with sensitive or emotionally charged topics (Harper, 2002).

Researcher-generated photo-elicitation, in which the researcher provides the photos for the participants to respond to (Richard & Lahman, 2015), can be useful when participants do not have access to cameras or are not comfortable taking their own photos (Collier & Collier, 1986). Furthermore, researcher-generated photos can be used to elicit specific information from participants that may be difficult to obtain through other means (Banks & Zeitlyn, 2015), such as insights into their daily routines, detailed accounts of experiences in specific environments, or emotions associated with particular settings. However, researcher-generated photos may not accurately represent the participants’ experiences or perspectives, which can lead to inaccurate or incomplete data (Pink, 2021). It has also been suggested that researcher-generated photos may not be as engaging or evocative as photos taken by the participants themselves (Harper, 2002). Thus, the method may not be as effective in triggering experiences, for example, memories and emotions, as photos taken by the participants themselves, leading to less rich or detailed responses. It is also important to consider that researcher-generated photos may be more difficult to interpret as the researcher’s own perspective may influence the choice of photos and in effect the participants’ responses (Banks & Zeitlyn, 2015).

The use of photo-elicitation in research has increased, and the method is now considered an important tool for gaining insights into experiences and perspectives of individuals (Church & Quilter, 2021). However, a limited number of publications detail the use of photo-elicitation as a standalone process (Bugos et al., 2014), potentially hindering the progress of studies utilizing this technique. This study seeks to fill a gap in the literature by reviewing transcripts of interviews that incorporate researcher-generated photos. Specifically, using data from a previous study (Kneck et al., 2022), we explore the use of researcher-generated photos during interviews about health with women experiencing homelessness. Guided by the following research questions, we aim to identify patterns in the interviews and provide insights for practical applications in future research, especially in underserved populations:

1. How did the women perceive the process of selecting photos?
2. How often does the interviewer/do the women refer to the photos?
3. When during interviews are the photos referred to?
4. Do the women use the photos to initiate a new topic or to continue a line of reasoning?
5. How are researcher-generated photos utilized in interview interactions, by the women and the interviewer?

## Method

This descriptive exploratory study is part of an interdisciplinary research program *Inclusion Health for Women Experiencing Homelessness* with the overall goal to address the health inequities that women in homelessness face. In accordance with the UK National Institute for Health Research (NIHR) Research Design Service [NIHR, 2021], an advisory board consisting of women with lived experience of homelessness contribute to the project. They actively contribute to project management, data analyses, interpretation, and dissemination of findings, alongside the researchers, working on equal terms as co-researchers and receive economic compensation in accordance with established guidelines for public contribution in research.

### Setting and Participants

In Sweden, more than 33 000 (National Board for Health and Welfare, 2017) citizens or individuals who have temporary or permanent residence permits are estimated to live in homelessness. The number of women in homelessness is increasing and constitutes approximately 40% of the homeless population, with a mean age of 39 years.

Women experiencing homelessness face a range of health and social issues that often are intertwined and mutually reinforcing. These include a high likelihood of domestic violence or trauma, leading to housing instability (Sullivan et al., 2023), and increased risk of mental health problems such as symptoms of depression, anxiety, and post-traumatic stress (Beijer et al., 2018). Women in homelessness may also struggle with substance use, both to manage the stresses of homelessness and the subsequent underlying mental health or trauma issues (Arnos & Acevedo, 2023; Beijer et al., 2018). Social isolation and feelings of being disconnected from family, friends, and community can exacerbate existing problems (Diduck et al., 2022). A comprehensive approach that includes overlap between healthcare, mental health support, and substance use treatment, and stable housing is necessary to address these needs (Omerov et al., 2019).

Data were collected via interviews in a primary healthcare center for persons in homelessness in Stockholm, Sweden, from December 2019 to January 2020. The center is open on weekdays and caters to persons in homelessness within the Stockholm Region, with a population of 2.4 million. Visits are free of charge, and the center has close collaborations with primary care, psychiatric care, services for treatment of substance use disorder, and social services. During 2019, approximately 1300 persons, 40% women, were cared for in the healthcare center, with 14,000 annual visits.

It is important to note that the interviews analyzed in this study build upon a prior research endeavor that aimed to describe reflections of health among women experiencing homelessness (Kneck et al., 2022). Researcher-generated photos were employed as stimuli to facilitate discussion. In this secondary analysis of photo use in research, we explore the utilization of photos during these interviews.

Participants were recruited in the waiting area of the center, using convenience sampling. One female researcher and one female research assistant were present in the waiting area, approached potential participants, and informed them about the study. The interviewer was a middle-aged female researcher with extensive experience of qualitative interviewing with persons with diabetes, women in the Roma population, and younger girls in sports clubs. She is trained in observational and visual methods in qualitative research (doctoral university course) and has used photo-elicitation in previous studies. She is a registered public health nurse and has not worked with women in homelessness previously. Her focus in conducting the interviews was to elicit reflections on health in homelessness from a holistic view, not limited to the absence of disease or infirmity but health as *a resource for living* (Kneck et al., 2022).

Inclusion criteria were Swedish-speaking women with experience of homelessness. The four categories of the European Typology of Homelessness and Housing Exclusion (ETHOS) (Busch-Geertsema et al., 2016) were used to define homelessness: roofless; houseless; insecure accommodation; and inadequate accommodation. Women exhibiting severe anxiety or distress in the waiting room, manifesting as violent or abusive behavior, were not approached. If a woman wanted to participate, oral and written information about the study was provided by the interviewer in an adjoining room, before the interview was conducted. Written informed consent was provided by all participants at the start of interviews. All background characteristics were self-reported. As a token of appreciation, the women received a gift certificate (20 euros) valid in a national chain of grocery stores.

Thirteen women agreed to participate in the study. The youngest woman interviewed was 30 years old and the oldest was 60 (median age 44 years). Time in homelessness varied between 13 days and 30 years. Two women could not specify how many years they had been in homelessness.

According to the ETHOS (Busch-Geertsema et al., 2016), the majority were defined as roofless (*n* = 5), that is, living in public space, or houseless (*n* = 5), that is, living in accommodation for people in homelessness. Three women lived in their own apartments, assigned through social services.

The women reported that they were suffering from substance use (*n* = 9); neurodevelopmental disorder (*n* = 5); chronic physical illness (*n* = 2); mental illness (*n* = 2); and intimate partner violence (*n* = 1). Six of thirteen women reported more than one illness/psychosocial problem.

### Data Collection

In this study, we used researcher-generated photo-elicitation to collect data (Harper, 2002). The decision to use this method was driven by safety and privacy concerns for the participants. While it is true that many individuals experiencing homelessness possess personal mobile phones (McInnes et al., 2013), we recognize the unique challenges they face. In previous interviews, women shared their experiences of being robbed of their mobile phones and feeling apprehensive about using them in public spaces due to risk of theft (Kneck et al., 2021). To address these concerns, the interviewer provided the photos for the interviews, using them as catalysts for discussions. This approach ensured that the women’s personal information, including their location or living conditions, remained confidential, and minimized the potential for harm and theft during data collection.

The interviewer had selected 20 photos each representing examples of everyday situations highlighting aspects of health and with possibilities to elicit feelings like belonging, hope, and love. Photos were selected from three anthologies of Swedish photography. After consenting to participate, the women were presented with the photos displayed on a table in the interview room. The interviewer encouraged the women to select five photos that made them feel good or that they associated with well-being. Interviews started by inviting the women to reflect or elaborate out loud about what they saw in the photos (*n* = 5) they chose. To deepen the dialogue, the interviewer used probes to encourage elaborations or to invite new topics. Interviews were audio-recorded pending consent and transcribed verbatim by a professional transcriber.

### Data Analyses

To illuminate how researcher-generated photos were utilized, we employed conversation analysis (CA), a sociological theory and method for studying interactions rooted in ethnomethodology (Garfinkel, 1991; Heritage, 1984). The underlying theoretical assumptions posit that linguistic utterances, coupled with non-verbal behavior, constitute communicative actions individuals undertake in interaction with others and their environment (Goodwin, 1995; Schegloff, 2007; Sidnell, 2010). CA researchers typically engage with small-scale phenomena, such as individual words or utterances, basing their analyses on detailed observations of what transpires in interactions. The objective is to discern the communicative actions performed, the resources (e.g., words or gestures) employed to carry out these actions, and how the actions interrelate. To achieve this, recorded interactions are scrutinized in detail, often employing meticulous transcriptions (see, e.g., Jefferson, 2004).

Verbal utterances not only serve to describe the world around us but also simultaneously influence the environment, as social situations evolve due to communicative actions (Heritage, 1984; Schegloff, 2007; Sidnell, 2010). One objective with the CA method is to describe these situated, communicative actions and their consequences. This entails highlighting communicative functions in individual utterances, understanding how they fit together, and recognizing how the interplay creates an arena for actions and utterances (Heritage, 1984; Schegloff, 2007). A question prompts a subsequent response, an offer engenders expectations of acceptance or rejection, and so forth.

A specific type of interviews studied within CA is the research interview (see, e.g., Roulston, 2006; Maynard et al., 2010). In most of these studies, the empirical material consists of interviews previously conducted by other researchers, in different fields and for different purposes, which CA researchers choose to re-examine. The crucial distinction lies in the purpose: The original researcher conducted the interview as a method to seek answers to something else, something beyond the interview situation itself, for example, to explore experiences, thoughts, and emotions of a specific group. The CA researcher emphasizes that a research interview must be regarded as its own social practice and endeavors to describe this practice, namely, to delineate the interactional patterns in the interplay between the interviewer and the interviewee.

The conversation analysis followed four broad stages: collecting-building, individual case analysis, pattern-identification, and evaluating patterns (Sidnell, 2010). The first author reviewed all audio files and transcriptions from the interviews (*n* = 13) and noted all cases where any of the participants or the interviewer referred to or alluded to a photo. To capture detailed information about the interaction, transcripts were elaborated during initial analyses to include information about pauses, overlapping speech, and other speech patterns. Thus, a transcription key was established to indicate the meaning of different symbols used in the transcriptions (Sidnell, 2010). For each case, the following information was noted: (i) the initiator of the conversation, whether it was the participant or the interviewer; (ii) the communicative act performed, analyzed using conversation analysis; (iii) the content of the conversation, including, for example, whether the woman talked about her personal experiences and described her feelings toward the image; (iv) based on the findings from (ii) and (iii), categories were established and compared quantitatively; and finally, (v) illustrative examples were analyzed with CA-methodology and selected to demonstrate patterns in the entire material. Findings and analyses were presented, discussed, and validated among authors in analytic research team meetings.

In addition, to answer research questions 1–3, interview characteristics were described, comprising length of interviews (minutes, median, and range), number of times photos were referred to (*n* (%), median, and range), and use of photos (*n* (%)) in relation to interview minutes (during the first 5 minutes, between 5 and 10 minutes, and after 10 minutes). To answer research question 3, the data regarding how the women used photos was grouped into two main categories: either initiation or continuation. Initiation pertained to when the women on their own initiative referred to one or more photos without being prompted to do so, while continuation entailed continuing a line of reasoning by referring to what was already an active part of the interview. The above-described steps in the analysis comprised a form of quantitizing data, assigning numerical values, to simplify and to enable pattern recognition, while preserving the complexity of the narratives (Sandelowski et al., 2009).

### Authors’ Profiles and Expertise

The first author, GB, is a male senior researcher in Scandinavian languages, bringing in extensive expertise in studying language and interaction through sociological methods (conversation analysis) and linguistic methods (interactional linguistics). His research encompasses a wide array of conversational context. Of particular interest is his focus on elucidating the social dynamics occurring when a researcher engages with an informant during a research interview. The second author, EM, is a female registered nurse/midwife, professor, and research-group leader with a wealth of experience in employing various qualitative research methods to explore the needs, distress, and preferences surrounding care. Her research primarily centers on equal healthcare for women experiencing homelessness, building on public contributions. The third author, LvE, is a female psychologist, professor, and research-group leader who possesses extensive expertise in both teaching and employing diverse qualitative research methods. Her work focuses on needs, distress, and preferences regarding psychological support among different groups of patients and carers in Sweden and Tanzania. Additionally, she has a considerable background in involving public contributors in her research and teaching about public contribution. The last author, AK, is a registered nurse and senior researcher with substantial experience in qualitative methods, specializing in studies involving women experiencing homelessness, patients and families in specialist palliative home care, and registered nurses. She is trained in qualitative interviewing and analyses.

### The Women Advisory Board

In Spring 2020, we established the Women Advisory Board, a collaborative reference group of women with lived experience of violence, abuse, and homelessness, working as co-researchers. This board serves two main purposes: to ensure and promote public contribution in our research program, for example, prioritizing research agenda items, and interpreting and presenting research findings. Board members convene weekly for 2-hour workshops with the second and last authors. The members receive compensation on an hourly basis through temporary employment at the university, in accordance with guidelines for public contribution and involvement. It is important to note that due to protected identities, most of the women cannot have their names published. Instead, we have collectively decided to refer to them as a consortium, the Women Advisory Board, in our publications.

During November and December of 2022, the Women Advisory Board, consisting of six women, participated in three workshops, totaling 6 hours, focused on interpreting the findings in this study. The goal was to enhance the relevance and applicability of the findings and thereby inform the development of practical applications in research contexts. The workshops included brainstorming sessions, group discussions, and individual reflections, with the aim of incorporating the experiences of women who have lived through homelessness. The second author and a research assistant attended the workshops with the women to provide support and facilitate the sessions.

During the first workshop, the second author introduced the photo-elicitation method used in the study, and the board members shared their thoughts of the findings in a brainstorming session. In the second workshop, the board members were provided with printed copies of the 20 study photos and were encouraged to reflect on them, take notes, and discuss their interpretations. During the third workshop, the board members summarized their interpretation of the study findings, considering the discussions and reflections from the earlier workshops. The research assistant took notes, and the resulting collective insights and perspectives of the Women Advisory Board were used to identify key topics for discussion and further exploration within the context of this article’s Discussion section.

## Ethics

The original interview study had ethical approval from the Swedish Ethical Review Authorities [Dnr: 2019-02130], and written informed consent proceedings were adhered to in accord with the Helsinki Declaration (World Medical Association, 2013). For the secondary analyses of data presented in this study, an additional ethical approval application was submitted and approved [Dnr: 2020-02457]. Due to the nature of the original study and the fact that the participants were women experiencing homelessness, it was not possible to obtain additional informed consent from the participants for the secondary analyses. However, all data were self-reported, and no personal identifiers or information from medical records were collected.

At the time of the original study (Kneck et al., 2022), the interviewer was unaware that data would be used for secondary analyses. However, this can be seen as a strength of the present study, as it ensured that the interviewer acted in accordance with the original study in the interviews. We informed the interviewer about the secondary analyses and obtained her approval for the use of the interviews for this purpose. The interviewer was not involved in the present study.

Turning to the study’s method, we believe that the use of researcher-generated photos was a responsible and ethical approach to collect data in this context. It minimized potential risks and prioritized the safety of the participants.

## Findings

### Selection of Photos

See Table 1 for an overview of photos and how many times each photo was chosen by the women.

**Table 1.** Description of Photos (*n* = 20) and Number of Times Each Photo Was Chosen.

Photo	Description	Number of times chosen
Women at a café	Two women sitting outside a busy café, drinking coffee. The women each have a small dog in their lap.	8
Baby	Naked baby sitting on the floor.	6
A man with two children	A man in bed looking at a gift. Two children joining him, one is jumping upside down and the other is sitting next to him.	6
Couple	A man and a woman sitting next to each other. The woman has her arms around his neck.	6
A residential area at night	A residential area at night, the heaven has a green color. A high-rise building is in the spotlight.	6
Bowling hall	A boy and a woman at a bowling hall. Both throw their balls.	5
Silhouette of a large tree	Silhouette of a large black tree against a yellow and orange background.	5
A woman in bed	A woman lying on her back with closed eyes on a bed. Behind her a large window with drawn curtains where the light sprinkles in.	4
People dancing	People in different ages shaking. A young smiling boy is in the forefront followed by an older man dancing with a couple of women.	3
A woman and a parrot	A woman in a sheer red dress and a red flower in her hair singing. Next to her a climbing parrot in an ornate steel stand.	3
A woman on a train platform	A woman squatting on a train platform with a laptop in her lap.	3
A woman reading a diary	A woman, looking into the camera, with a diary in her lap and a cup of coffee.	3
An older woman with punk hairstyle	An older woman with punk hairstyle wearing sunglasses, a necklace with metal rivets and headbands.	2
People sitting in front of a cottage	People sitting in front of a cottage in rain with raised umbrellas. Their backs are turned against the camera.	2
A woman lying on a hospital bunk	A woman lying on her back on a hospital bunk.	1
Four women in a formation	Four women standing in a close formation. Three women are dressed in black, and one woman is naked.	1
Young woman on a skateboard	A young woman sitting on a skateboard looking at her mobile.	1
A woman with a helmet	A woman with a yellow helmet standing in a tunnel.	0
A woman looking up in the sky	A woman wearing a polka dot t-shirt, looking up in the sky with arms raised touching her own neck. She is standing against a colorful wall with dots.	0
Two women in front of a pedestrian crossing	Two women standing with their backs against the camera in front of a pedestrian crossing.	0

The photo chosen by most, eight, women pictured women sitting outside a café drinking coffee. Three photos were never chosen.

Selection of photos was introduced prior to the interviews. This introduction did not seem to influence whether a participant agreed to participate, and all women who consented to participate were willing to engage with the task. Based on audio files and transcriptions of the interviews, the women generally understood the task and thoughtfully engaged with it. They actively selected photos that resonated with them and provided explanations for their choices, as illustrated with the following excerpt from interview 6:

Woman: Mm ... yeah, that ... hmm ... (short pause). That one. And that one. 1, 2, 3, 4, 5 [Chosen photos: women at a café, baby, a man with two children, couple, and bowling hall, see Table 1].

Interviewer: Mm, let’s see ... And then if you look at those five photos that you’ve chosen now, in terms of feeling good.

Woman: Yes, it’s the family. Regardless of how it is, you know. Yes, you can probably say that, that you have someone you can share everything with, yes.

Interviewer: Yes, tell me a little about what you see in the photos.

Woman: Well, I only see like ... love, you know, that you have someone to lean on when it’s tough and all that. And then it’s ... it’s community, and it doesn’t matter if it’s rainy, as long as you have a good time together and like this.

In this excerpt, the woman provides a detailed explanation for her choices of photos, emphasizing the significance of family and the sense of togetherness and support it brings to her well-being.

However, there are some examples that suggest participants may have hesitated or needed guidance in the selection process. In interview 5, the woman initially hesitates before making her selection:

Woman: Yes, now, I’m not sure if this has to do with health but it …

Interviewer: Mm … (short pause).

Woman: Well, I think those ones.

In this excerpt, the woman expressed uncertainty about whether her choice of a photo is related to her perceptions of health. After a brief pause from the interviewer, she ultimately makes her selection.

### Interview Length and Frequency of Photo Reference in the Interviews

The total number of times photos were referred to in each interview, by the women (*n* = 13) and the interviewer, respectively, and interview length are presented in Table 2. To facilitate readability, the interviews are sorted in descending order according to total number of times.

**Table 2.** Number of Times Photos Were Referred to in Total, by the Women and the Interviewer, Respectively, and Interview Length.

Interview, number	Total number of times	Women (*n* = 13) number of times	Interviewer number of times	Interview length (minutes)
11	20	11	9	16
2	18	9	9	35
8	13	5	8	28
5	13	6	7	22
1	10	2	8	29
9	9	4	5	38
4	9	2	7	30
13	7	4	3	66
3	6	0	6	20
10	5	2	3	19
7	3	0	3	34
6	3	0	3	27
12	2	0	2	10
Total	**118**	**45**	**73**	**374**

The interviews lasted between 10 and 66 minutes, and the median interview length was 28 minutes. During the total interview length, 6 hours and 23 minutes, the photos were referred to 118 times (median 9, range 2–20) by the interviewer and the women. The interviewer referred to the photos 73 times (62%, median 6, range 2–9), whereas the women referred to the photos 45 times (38%, median 2, range 0–11). In two interviews, the women referred to the photos 11 and nine times, respectively, whereas the interviewer in the same interviews referred four and three times to the photos. These interviews lasted between 16 and 66 minutes. Four women did not refer to the photos at all. These interviews lasted between 10 and 34 minutes.

### Usage Patterns of Photos During the Interviews

The number of times photos were referred to in each interview during the first 5 minutes, after 5 to 10 minutes, and after 10 minutes by the women (*n* = 13), the interviewer, and in total are presented in Table 3.

**Table 3.** Number of Times Photos Were Referred to During the First 5 Minutes, After 5 to 10 Minutes, and After 10 Minutes.

Number of times photos were referred to during the interview									
Interview, number	Women (*n* = 13)			Interviewer			Total		
	First 5 minutes	>5–≤10 minutes	>10 minutes	First 5 minutes	>5–≤10 minutes	>10 minutes	First 5 minutes	>5–≤10 minutes	>10 minutes
11	10	1	0	7	2	0	17	3	0
2	5	3	1	3	4	2	8	7	3
5	4	1	1	7	0	0	11	1	1
8	3	0	2	4	1	3	7	1	5
9	1	3	0	0	3	2	1	6	2
13	0	2	2	3	0	0	3	2	2
1	2	0	0	5	1	2	7	1	2
4	0	1	1	4	2	1	4	3	2
10	0	0	2	2	1	0	2	1	2
3	0	0	0	3	3	0	3	3	0
7	0	0	0	2	1	0	2	1	0
6	0	0	0	3	0	0	3	0	0
12^a^	0	0	-	2	0	-	2	0	-
**Total**	**25**	**11**	**9**	**45**	**18**	**10**	**70**	**29**	**19**

^a^Length of the interview = 10 minutes.

During the first 5 minutes of the interviews, the photos were referred to 70 times (median 4, range 2–17). The women referred to the photos 25 times (36%, median 0, range 0–10) and the interviewer 45 times (64%, median 3, range 2–7). In one interview, the interviewer did not refer to the photos at all, while in seven interviews, none of the women referred to the photos during the first 5 minutes.

Later in the interviews, after 5 to 10 minutes, the photos were totally referred to 29 times (median 1, range 0–7). The women referred to the photos 11 times (38%, median 0, range 0–3) and the interviewer 18 times (62%, median 1, range 0–4). During four interviews, the interviewer did not refer to the photos in this time segment, while the women did not refer to the photos in seven interviews of the same time segment.

After 10 minutes of interview time (12 interviews), the photos were referred to 19 times (median 2, range 0–5). The women referred to the photos nine times (47%, median .5, range 0–2) and the corresponding numbers were 10 times (53%, median 0, range 0–3) for the interviewer. In six interviews, the women did not refer to the photos, and in seven interviews, the interviewer did not refer to the photos.

### The Women’s Patterns of Use of Photos in Initiating and Continuing Topics During the Interviews

How frequently the women (*n* = 13) used the photos to initiate a new topic or continue a line of reasoning during the interviews is presented in Table 4. To facilitate readability, the interviews are sorted in descending order, that is, the interview with most referrals to the photos to initiate a new topic is reported first in the table.

**Table 4.** Number of Times the Women (*n* = 13) Used the Photos to Initiate a New Topic or Continue a Line of Reasoning During the Interview.

Interview, number	Initiation number of times	Continuation number of times
9	4	0
11	2	9
2	2	7
5	1	5
8	0	5
13	0	4
1	0	2
4	0	2
10	0	2
3	0	0
6	0	0
7	0	0
12	0	0
Total	9	36

Photos were used 36 times (80%) by the women to continue a line of reasoning and nine times (20%) to initiate a new topic. Eight women used the photos to continue a line of reasoning by referring to photos that were already an active part of the interview, whereas four women used the photos without being prompted to do so. Interview number 9 with four initiations is an exception, and the woman assertively “assumes control” of the interview situation by talking about the photos she has chosen. This interview comes across as more of a monologue, with the woman sharing disturbing/distressing experiences without being prompted and without follow-up questions from the interviewer.

### Utilization of Researcher-Generated Photos in Interview Interactions: Patterns From the Women and the Interviewer

The conversation analysis of the interviews illustrated that the women referred to the photos in three ways: (1) as drivers of associations and emotions, (2) to describe the content of the photos, or (3) the photos did not have any prominent role during the interview. To illustrate this breadth, three interviews were selected. The findings are presented below, starting with interview 5. In this interview, the woman focused on interpreting the photos by reflecting on what the photos symbolized to her and the emotional responses they evoked. In interview 11, on the other hand, the woman focused on describing the content of the photos using concrete language. Finally, in interview 13, the longest of the interviews, the woman elaborated on her narrative in depth but with sparse use of the photos.

Starting with interview 5, an elaborated transcription illustrates an episode where photos function as drivers to facilitate further reflections (see Table 5).

**Table 5.** Elaborated Transcription Interview 5 (After 5 Minutes).

Line	Interviewer (I) or participant (P); utterances
01	I: but if you think that you have this a little ( . ) you have a bed and can lie
02	down listening to [music
03	P: [yeah got an apartment [er ( . ) and it’s so ( . ) [er
04	I: [hmm [hmm
05	I: hmm
06	P: and then that I like to perform ( . ) I like to clown around
07	a little bit ( . ) like I’m used to having this this ( . )
08	midsummer with family and ( . ) and dancing and stuff like ( . ) and have love that’s
09	that’s like er ( . ) my old life now it’s more ( . ) like this ((probably points
10	to another image)) ( . ) or I don’t know ((laughing))
11	I: I see so what do you think
12	P: nuh but like that you dare to do something ( . ) perform or
13	I: hmm
14	P: well talking in front of an audience I’ve sort of I’ve ( . ) played theater
15	I’ve been in movies like ( . ) you dare much more
16	I: hmm
17	P: or you don’t dare but you still do it
18	I: ok
19	P: and when you’ve done it you feel good much better
20	I: ok
21	P: hmm
22	( . . )
23	I: so you think you can ( . ) you challenge yourself more now ya think
24	P: yes I do much more
25	I: hmm
26	P: I always say like this ( . ) I survived that ( . )
27	that ( . ) that ( . ) that is much worse than anything else
28	I: hmm
29	P: that’s how it is
30	I: hmm
31	P: I can’t can’t can’t make a fool of myself all life long but ( . ) think of
32	what I’d done if I’d been doing drugs on the street [like ( )
33	I: [hmm
34	I: hmm
35	P: so that would have been dangerous so ( . ) to me it’s like
36	that I dare to do more things that I didn’t before
37	I: hmm ( . ) and that makes you feel good
38	P: yes ( . ) it really does
39	((I asks a question about another image))

Transcription key: ( . ) = a pause shorter than 1 s; ( . . ) = a pause longer than 1 s; (( )) double parentheses = comment from researcher; ( ) empty parentheses = something is said but not distinguishable; [ = overlapping speech.

In lines 1–2, the interviewer summarizes what the woman has said so far about the photo (Table 1: woman in bed) and seeks to validate her own understanding of the woman’s statements. The way the question is stated discourages a simple yes/no answer and encourages the woman to continue. She mentions positive change in her life: that she has her own apartment and has moved from being a person experiencing substance use disorder and living on the street (line 3), which has resulted in her feeling much better in general (lines 27, 31, 32, 35, and 36). Also, she shares a change over time that is both for good and bad, from a safe life near a loving family (lines 7–9) to a more uncertain existence where the family has removed themselves; but on the other hand, she has found new courage in performing music in front of others (lines 6, 7, 12, 14, 15, 17, and 24). This makes her feel good (lines 19 and 38). As we understand it, her life is divided into three chronological phases: (1) a normal, safe life, (2) a destructive life of a person experiencing substance use disorder and struggling with a destructive life on the streets, and (3) her present life with a home and meaningful activities, but without safety and love from family. Taken together, in interview 5, the photos seem to have a crucial role as drivers of associations and emotions to describe health and well-being, in interaction with the interviewer.

In interview 11, the photos are referred to repeatedly, but the time spent discussing each photo is brief and descriptions are concrete. In the elaborated transcription, three photos are chosen by the woman: (1) a silhouette of a large tree, (2) a bowling hall, and (3) a man with two children (Table 1). Table 6 exemplifies a conversation analysis transcription of an episode in interview 11 that is representative for the interview in its entirety.

**Table 6.** Elaborated Transcription Interview 11 (After 3 Minutes).

Line	Interviewer (I) or participant (P); utterances
01	I: tell me more
02	( . . )
03	P: well er (.) you mean the northern lights
04	I: hmm
05	P: I’ve seen it a couple of times
06	( . )
07	I: hmm
08	( . )
09	P: well then ( . ) like I said yes the moon the moon plays into this too
10	I: hmm ( . ) hmm
11	( . . )
12	P: actually so that ( . ) you get sort of high in another way sort of ( . )
13	You go skiing or something huh
14	I: hmm
15	P: that can get you high too
16	( . )
17	I: ok
18	P: the adrenaline and serotonin and stuff
19	I: yes
20	( . . )
21	P: so I think ( . . ) in that way ((seems to point
22	to another image)) ( . ) [ ( )
23	I: [ the bowlers
24	P: yeah that’s ( . ) nice sport ( . . ) but that dad who’s
25	tired is a bit annoying
26	(( referring to a new image; said with laughter))
27	I: ok [ ( )
28	P: [( )
29	( . )
30	P: this is nice ((seems to return to previous
31	image above)) ( . . ) too ( . ) it crackles
32	( . )
33	I: what do you see in the image then
34	P: that it crackles in in ( . ) up in ( . ) one of those ( . . )
35	what shu ma call it ( . . ) northern lights ( . ) [it crackles
36	I: [hmm
37	I: ok
38	P: have you seen that anytime
39	I: hmm
40	P: er ( . ) it crackles like this sch ((makes noise illustrating crackling))
41	I: hmm
42	P: but it’s something like that ( . . ) hmmm
43	I: the tree ( . ) hmm
44	P: hmm
45	I: what in the light makes you feel good then
46	P: the light
47	( . )
48	I: hmm
49	P: hmm
50	( . )
51	I: in what way
52	( . . )
53	P: it’s like ( . ) peoples ( . ) if you are colorblind
54	that makes it hard huh ((briefly described how different colors affect her))

Transcription key: ( . ) = a pause shorter than 1 s; ( . . ) = a pause longer than 1 s; (( )) double parentheses = comment from researcher; ( ) empty parentheses = something is said but not distinguishable; [ = overlapping speech.

Before the interviews started, the women were encouraged to select photos that made them feel good or that they associated with well-being. However, in interview 11, the woman limits her description to what the photo portrays, without referring to her personal life, emotions, or opinions. The woman has been asked to *share what she sees in the photos*. It may be noted that the wording leaves ample room for interpretation, either in a literal and objective manner or a more abstract manner. The woman chooses a photo with a silhouette of a large tree and a light phenomenon, stating that she and others *get high* from the northern lights and moonlight. There are no explanations and/or elaborations in relation to health and well-being in the context of being a woman in homelessness.

The woman mentioned that she likes bowling (line 24) but nothing about how or why. Also, she reacts negatively to the man with two children whom she interprets as being tired (lines 24–25); however, she conveys nothing specific about the negative associations. Finally, when she returns to the photo with the silhouette of the tree and the light, we receive an imitation of the sound (line 40), and she says that the sound makes her feel good (line 46). It is worth noting that the woman also poses a counterquestion (line 38). It occurs several times in this interview but is rare in the other interviews. The counterquestion can presumably indicate that she has nothing further to say about the topic nor that she has any interest in discussing it further. Counterquestions may be regarded as challenging in themselves, since the norm stipulates that one person in an interview situation poses questions, and the other is expected to answer. Taken together, although some photos are referred to repeatedly in interview 11, they are not used to elaborate descriptions on health and well-being.

To explore our findings further, we analyzed interview 13 similarly to interviews 5 and 11. In this interview, the woman provided a detailed and comprehensive narrative. The woman reflects on her life, describes her feelings and goals for the future, and potential obstacles to reach her goals. A large proportion of the reflections pertains to challenges associated with being a woman, and she emphasizes that the situation for women in homelessness is significantly different from that of men. All this is done in interaction with the interviewer, without the photos having any prominent role, and several topics are introduced by the woman, as opposed to providing answers to interview questions. Taken together, in interview 13, the woman influences and governs the interview situation in interaction with the interviewer. The photos seem to have a secondary or minor role in achieving this.

Turning to the interviewer, we conclude that she acts in a similar manner in interview 5 as in interviews 11 and 13. In the interviews, she produces several encouraging signals to the women to continue (e.g., Table 5: lines 4, 5, 13, 16, 18, and 20) and poses follow-up questions that signal interest and provide the women possibilities to continue (Table 5*: what are you thinking*, line 11; *you challenge yourself more*, line 23; *that makes you feel good*, line 37). In interview 5, it is more effective, since the woman shares her own experiences, thoughts, and feelings rather than describing what is visible in the photos.

## Discussion

### The Researchers’ Reflection on the Findings

Overall, the participants did not engage extensively with the photos during the interviews, particularly after the initial moments. The conversation analysis revealed three primary ways in which the women interacted with the photos: factual descriptions of the content, eliciting associations and emotions, and a more peripheral role in the interviews. These patterns are hard to interpret, and a recent review highlights a challenge in assessing the impact of visual methods. It is complex to isolate their contribution from factors like relationships, language-based communication, and the interviewer’s approach (Pain, 2012). In our study, the interviewer and the participants had no prior relationship; they met for the first time. Consequently, they needed to build rapport as the interview progressed. This may explain why photos were utilized most during the initial minutes of the interview, potentially reflecting a slight initial tension from both the interviewer and the women. As observed in previous studies (Harper, 2002), the photos seemed to have served as icebreakers.

The observed patterns in the women’s engagement with the photos may be attributed to the inherent versatility of the method, allowing for a range of interpretations and responses (Richard & Lahman, 2015). However, it is crucial to recognize that these patterns could also signify the complexity of the method, particularly within the context of women experiencing homelessness. Factors such as trauma, mental health challenges, and substance use disorder (Aldridge et al., 2018; Fazel et al., 2014), coupled with experiences of stigma and discrimination (Kneck et al., 2021), may introduce additional layers of complexity, influencing their interactions with their surroundings and their responses to visual stimuli. Therefore, the identified patterns may represent a combination of the method’s versatility (Richard & Lahman, 2015) and its potential challenges (Banks, 2007), underscoring the importance of considering the unique circumstances of this population in visual research methodologies.

The observed patterns from the interviews indicate that the interviewer predominantly referred to the photos, while the women did not utilize them to assert their voice or choice, such as in initiating new topics. It may be inferred that the differences in photo referrals between the interviewer and the women are influenced by our use of researcher-generated decontextualized photos—a set of photos not directly related to the research participants, topics, or setting. This approach has been noted for its potential challenge in maintaining interview focus due to the absence of contextual ties (Banks, 2007). Furthermore, this underscores the ongoing discussion regarding the utilization of researcher-generated photo-elicitation methods. There is a concern that such methods may unintentionally prioritize researchers’ perspectives over those of the participants, potentially reinforcing cultural stereotypes (Copes et al., 2018).

On the other hand, previous reasearch has recognized the potential benefits of utilizing decontextualized photo sets. This method not only complements the advantages of photo-elicitation interviewing but also provides researchers with a less time-consuming and logistically simpler option than participant-generated photo-elicitation (Richard & Lahman, 2015). These considerations are particularly crucial in research with hard-to-reach populations. Furthermore, despite the lack of specific contextual constraints, decontextualized photos have the potential for the research question/s to serve as the guiding context (Richard & Lahman, 2015).

Our study involved 13 participants and one interviewer, which limits our ability to draw conclusions about whether researcher-generated photos have the potential to give marginalized groups a voice and choice in research interviews. Nonetheless, the emerging patterns emphasize the importance of additional methodological studies exploring the potential and drawbacks of using researcher-generated photo-elicitation in interview situations. Furthermore, it is imperative for future studies to critically explore the nuances and implications of researcher-generated photo-elicitation within interview settings, considering factors such as participant rapport, power dynamics, and potential biases that may influence the elicited visual data. Method descriptions of visual methods in research tend to state positive outcomes of using visual methods. Elaborating nuances, implications of contextual factors, and potential bias may contribute to more robust studies, with respect for the inherent complexity in the research situation. Previous research has underscored the significance of establishing a supportive, empowering, and respectful environment (Seitz & Strack, 2016). By recognizing and addressing the women’s psychological needs for autonomy, competence, and relatedness (Phipps et al., 2021), researchers need to cultivate inclusive study designs and execution that authentically reflects the experiences and perspectives of women in homelessness.

### The Women Advisory Board’s Reflection on the Findings

The Women Advisory Board’s reflections regarding the participants’ use of the photos in the interviews highlight the importance of considering the participants’ perspectives and experiences when designing methods for researcher-generated photo-elicitation. The board’s unanimous opinion that the photos did not engage or stimulate the participants indicates a potential gap between the researchers’ intentions and the participants’ needs. The board highlighted three problematic areas: use of ambiguous researcher-generated photos, the interview location, and providing interview incentives with vouchers. The continued discussion will elaborate these points further.

The Women Advisory Board provided examples of ambiguous photos that could evoke conflicting emotions, for example, the photo of a residential area at night that six participants chose. According to the board, this could induce feelings of longing for the shelter of a home, alongside memories of violence, abuse, and rape in a residence. This underscores the importance of acknowledging that a photo can evoke a variety of responses. Another photo, picturing women sitting and drinking coffee outside a café, chosen by eight participants, was described as showing friendship and an ordinary life. However, according to the board members, this may be distressing due to the unattainability in their current situation of homelessness. This example highlights a major challenge with researcher-generated photos (Banks, 2007; Wang & Burris, 1997) and emphasizes the importance of considering the participants’ current living situation and how it may shape their interpretation of the photos. An approach of using both researcher-generated and participant-generated photos has been suggested to promote collaborative engagement and balancing perspectives better (Copes et al., 2018). The Women Advisory Board’s recommendation was to allow the women to take their own photos or choose photos in magazine for a more participatory approach that potentially could empower the women to express themselves more freely, for example, regarding their health and well-being. However, the safety concerns of having a valuable camera in uncertain circumstances stress the need of balancing the participants’ agency and autonomy with protection and safety.

The Women Advisory Board also provided valuable insights on the interview location. Four out of six board members had experiences of visiting the healthcare center where the interviews took place. While the interviews were conducted in an adjoining room and not in the waiting room, the board members still emphasized that the location was inappropriate and distressing for the participants and stressed that conducting the interviews in a more relaxed and confidential environment, such as a café, would have been more appropriate. This was contrary to our thoughts (as researchers) of cafés being more unsheltered and less private. Being in a safe space is considered necessary to establish rapport between the interviewer and the participant (Patton, 2015), and often crucial for building trust and facilitating open communication. Overall, the board’s feedback highlights the importance of considering the interview location from the perspective of the participants. It may also serve to illustrate the complexity of understanding the participants’ experiences and perspectives, as in our case when we did consider location with the participants in mind, and nonetheless decided on a less than ideal interview location.

The final issue raised by the Women Advisory Board was the use of incentives, a 20-euro gift card, to encourage participation in interviews. While the board agreed with the importance of acknowledging and compensating participants for their time and effort, they felt that this approach could be seen as buying participation. Moreover, the board members expressed concerns that some women may have consented to be interviewed simply to receive the voucher rather than to share their experiences. Other researchers describe trying to find a non-coercive amount as compensation or providing small favors like offering a ride or buying lunch (Copes et al., 2018). For women in homelessness, even a small amount may act coercively. The board was concerned that interview responses were not genuine and that participants may try to figure out what the researcher wants to hear to quickly finish the interview and receive the voucher. This in turn, may explain our participants’ scant use of the photos during the interviews. The board members emphasized the importance of respecting the participants’ agency and autonomy and that a more respectful approach would be to offer participants a choice of whether to receive a gift card or another form of compensation. Additionally, they reiterated the need to make sure that the compensation is not perceived as coercive or exploitative.

### Implications and Future Directions

This study represents a novel attempt to explore patterns of use of researcher-generated photos in interviews with a marginalized, underserved group in society. Our findings suggest that the approach can be useful to elicit participants’ perspectives and experiences. However, the Women Advisory Board recommended careful consideration to the selection of photos, and that the use of abstract pictures with colors and photos from nature may be particularly useful in eliciting participants’ own responses, as opposed to pleasing researchers to receive the promised voucher or gift card for participation. Additionally, the board members emphasized that the cultural and social context of the photos must be considered, since unintended associations may be evoked. As researchers, we would like to add our voices to the board’s, highlighting the importance of considering the selection of photos, the interview location, and the use of incentives in research with underserved, hard-to-reach populations. This way, we may engage and collaborate with underserved communities in a more respectful and ethical manner.

## Conclusion

This study found that researcher-generated photos can be a valuable tool for eliciting descriptions of health and well-being from women in homelessness. However, the findings also highlight the importance of considering the perspectives and experiences of the intended interview population, when choosing the photos. The interviews provide few examples of the activity, engagement, and initiatives described in photo-elicitation method literature, underscoring the complexity of conducting research interviews with underserved populations. The Women Advisory Board’s feedback provides valuable insights into critical considerations for the use of researcher-generated photos, such as the importance of choosing appropriate photos, considering the interview location, and reflecting on the use of incentives in research with women experiencing homelessness.

The board’s recommendations propose a more participatory approach to research design and implementation to empower participants to express themselves freely. Additionally, the board’s feedback underscores the importance of creating supportive and safe environments for interviews with women experiencing homelessness. These findings may inform future research on the use of researcher-generated photo-elicitation with underserved, hard-to-reach populations.
